# Quantification of Macular Vascular Density Using Optical Coherence Tomography Angiography and Its Relationship with Retinal Thickness in Myopic Eyes of Young Adults

**DOI:** 10.1155/2017/1397179

**Published:** 2017-11-29

**Authors:** Shiqi Yang, Minwen Zhou, Bing Lu, Pengfei Zhang, Jingke Zhao, Mei Kang, Ruoshi Wang, Fenghua Wang, Xiaodong Sun

**Affiliations:** ^1^Department of Ophthalmology, Shanghai General Hospital (Shanghai First People's Hospital), Shanghai Jiao Tong University School of Medicine, Shanghai, China; ^2^Clinical Research Center, Shanghai General Hospital (Shanghai First People's Hospital), Shanghai Jiao Tong University School of Medicine, Shanghai, China; ^3^Shanghai Engineering Center for Visual Science and Photomedicine, Shanghai, China; ^4^Shanghai Key Laboratory of Fundus Diseases, Shanghai, China

## Abstract

**Purpose:**

To quantify macular vascular density using optical coherence tomography angiography (OCTA) and to investigate its relationship with retinal thickness in myopic eyes of young adults.

**Methods:**

In this cross-sectional study, 268 myopic eyes without pathological changes were recruited and divided into three groups: mild myopia (*n* = 81), moderate myopia (*n* = 117), and high myopia (*n* = 70). Macular vascular density was quantified by OCTA and compared among three groups. Average retinal thickness, central subfield thickness, and macular ganglion cell complex (mGCC) thickness were also evaluated and compared. Correlations among these variables were analyzed.

**Results:**

There was no statistical difference in superficial (62.3 ± 5.7% versus 62.7 ± 5.9% versus 63.8 ± 5.5%) and deep macular vascular densities (58.3 ± 9.6% versus 59.2 ± 9.3% versus 60.9 ± 7.9%) among mild-myopia, moderate-myopia, and high-myopia groups (both *P* > 0.05). Superficial and deep macular vascular densities both had correlations with mean arterial pressure. Furthermore, superficial macular vascular density was significantly correlated with mGCC thickness.

**Conclusions:**

Varying degrees of myopia did not affect macular vascular density in young healthy adults. In addition, superficial macular vascular density, as an independent factor, was positively correlated with mGCC thickness.

## 1. Introduction

Myopia is one of the most prevalent eye disorders and is estimated to affect 1.5 billion people worldwide [1, 2]. With the rapid development of internet and information technology, recent epidemiological studies have reported a tremendous increase in the prevalence of myopia, especially in Asian countries, where reportedly 69–90% of school graduates are affected [3–11]. As a potentially sight-threatening condition, myopia has numerous visual and financial effects that can result in an impaired quality of life, reduced competitiveness for employment, and additional costs associated with visual aids. Furthermore, high myopia is a risk factor for several pathologies of the eye, including chorioretinal atrophy, myopic maculopathy, glaucoma, and other vision-threatening conditions [6]. Therefore, myopia is currently considered a major public health challenge.

Morphological changes of the vasculature system in myopic patients have been observed since 1977 [12]. However, studying the ocular vasculature previously has been a challenge due to several limitations in imaging modalities. For example, fluorescein angiography (FA) is an invasive examination that cannot be accurately quantified [13] and Doppler imaging mainly focuses on large vessels, rather than microvasculature [14, 15]. The recent development of optical coherence tomography angiography (OCTA) makes it possible to perform measurements of the retinal vasculature in a noninvasive manner [16, 17]. OCTA with split-spectrum amplitude-decorrelation angiography (SSADA) evaluates vascular density with high intervisit repeatability and reproducibility [18, 19]. Furthermore, OCTA with integrated software provides morphological information and quantitative measurement of the retinal vasculature system, including both large retinal vessels and the microvasculature [20–26], which can extend our understanding of retinal blood flow in myopia.

To characterize retinal blood flow and evaluate early changes in myopia, we aimed to quantitatively assess the macular vascular density in myopia of young adults with normal visual acuity. A further aim was to provide a broader understanding of the associations between macular retinal perfusion and retinal structure, which could contribute to identifying predictive patterns for the development of early-stage, myopia-related complications.

## 2. Materials and Methods

### 2.1. Study Subjects

This cross-sectional study was approved by the Ethical Review Committee of the Shanghai General Hospital affiliated with Shanghai Jiao Tong University and adhered to the provisions of the Declaration of Helsinki for research involving human subjects. All participants were informed of the purpose of the research and provided written informed consent prior to entering the study. All subjects were from a Han Chinese population and were recruited from June 2015 to July 2015 in Shanghai General Hospital.

All subjects underwent a comprehensive ophthalmologic examination, which included slit-lamp biomicroscopy, best-corrected visual acuity (BCVA), refractive error examination (AR-310A; Nidek, Japan), intraocular pressure (IOP) measurement (TX-200 tonometer; Canon, Japan), fundus examination, axial length (AL) measurement (IOL Master; Carl Zeiss, Germany), and central corneal thickness (CCT) measurement (Pachymetry SP-3000; TOMEY, Japan). Demographic information and medical history were recorded for all subjects, and their heart rates (HR) and blood pressures (BP) were measured at the time of the OCT imaging.

Subjects were included if they met the following criteria: between the ages of 18 and 32, BCVA equal to or better than 20/20, spherical equivalent (SE) ≤ −0.50 D, and normal IOP. The exclusion criteria were any ocular disorders except ametropia, any systemic diseases, a history of intraocular surgery or ocular trauma, a history of posterior segment laser treatment, a history of ocular hypertension or glaucoma, signs of myopic degeneration or a pathologic form of myopia, an inability to tolerate OCTA, and poor-quality images.

### 2.2. Optical Coherence Tomography Angiography Image Acquisition and Processing

In the OCTA system (RTVue-XR Avanti; Optovue, Inc., Fremont, CA, USA), AngioVue applied SSADA to detect vessels with blood flow through the intrinsic motion contrast provided by the flowing erythrocytes, making it possible to noninvasively obtain three-dimensional mapping of the retina and choroidal microvasculature [18, 19]. During scanning, two B-scans are captured at each fixed position, while two orthogonal OCTA volume scans (horizontal and vertical) are used to minimize motion artifacts and fixation changes. To evaluate macular vessels, a 3 mm × 3 mm scanning area centered on the macula was acquired and automatically divided into two segments, including the superficial and deep capillary plexuses [27, 28]. The superficial capillary plexus en face image was segmented with an inner boundary set at 3 *μ*m beneath the internal limiting membrane (ILM) and an outer boundary set at 15 *μ*m beneath the inner plexiform layer (IPL), while the deep capillary plexus boundaries were set at 15 to 70 *μ*m beneath the IPL. Quantitatively, superficial and deep vascular density (%) measurements were automatically calculated as the percentage of the measured area occupied by flowing blood vessels by the OCT machine's inner software (version 2014.2.0.93). All subjects were examined and assessed by two authors independently. Poor quality images, defined as those with a signal strength index less than 40 or registered image sets with residual motion artifacts, were excluded.

### 2.3. Optical Coherence Tomography Measurements

All subjects were examined with an RTVue-100 Fourier-domain OCT instrument (Optovue Inc., Fremont, CA, USA) using the scanning protocol “GCC” (ganglion cell complex). The FD-OCT system imaging was performed as described in previous studies [29]. Briefly, the macular GCC (mGCC) scanning protocol consisted of a 7 mm × 7 mm area, which measured the retinal thickness from the ILM to the IPL posterior boundary. The average mGCC thickness measurements were used for analysis. Two authors examined and assessed the image quality independently. Images of poor quality, defined as those with a signal-strength index less than 40, were excluded from the analysis.

The retinal thickness of the macular area was measured by Cirrus HD-OCT (Carl Zeiss Meditec, Dublin, CA, USA) using a macula cube 512 A-scans × 128 B-scans protocol. Full retinal thickness was measured from the ILM to the outer boundary of the retinal pigment epithelium (RPE). Eye movements were monitored by reading the real-time fundus images. We obtained average retinal thickness measurements in 9 subfield regions, according to the Early Treatment Diabetic Retinopathy Study (ETDRS). Average retinal thickness and central subfield thickness were used for the analysis. Two trained ophthalmologists examined and assessed the image quality independently. Images of poor quality, defined as those with a signal-strength index less than 7, were excluded from the analysis.

### 2.4. Statistical Analysis

The data were processed and analyzed with SPSS for Windows 8.0 software (Version 19.0; SPSS, Chicago, IL). The distribution of numeric variables was assessed by inspecting histograms and using Shapiro-Wilk *W* tests of normality. Numeric data are presented as the mean ± standard deviation (SD). One-way analysis of variance (ANOVA) was used for intergroup comparisons of normally distributed variables among the three myopia groups. Pearson correlation coefficients (*r* values) were used to investigate the associations between macular vascular density and clinical factors, including age, IOP at imaging, SE, pulse pressure, HR, mean arterial pressure (MAP), AL, and CCT. The relationship between macular vascular density and retinal thickness was analyzed through univariate linear regression. Variables with probability *P* < 0.05 were then included in multivariate regression analysis using a stepwise method. *P* < 0.05 was considered statistically significant for all analyses.

## 3. Results

Of all the recruited subjects for this study, ten eyes were excluded due to poor fixation and inferior-quality images. In total, 268 eyes from 145 subjects (59 male and 86 female) were analyzed in this study. The mean age of the subjects was 26.0 ± 1.7 years, and the mean SE of the subjects was −4.42 ± 2.19 D. These eyes were subsequently divided according to their degree of myopia: 81 subjects were included in the mild-myopia group (−0.50 D < SE ≤ −3.00 D), 117 subjects were included in the moderate-myopia group (−3.00 D < SE ≤ −6.00 D), and 70 subjects were included in the high-myopia group (SE< −6.00 D). The demographic and clinical characteristics of the subjects were summarized and compared in Table 1. The three groups were similar in terms of their mean age, IOP at imaging, systolic BP, diastolic BP, MAP, pulse pressure, HR, and CCT (all *P* > 0.05; Table 1).

The superficial macular vascular densities in the mild-myopia (62.3 ± 5.7%), moderate-myopia (62.7 ± 5.9%), and high-myopia groups (63.8 ± 5.5%) were not significantly different (*P* = 0.254; Table 2). Similar results were noted with deep macular vascular densities among the three groups (*P* = 0.202; Table 2). The results showed that average mGCC thicknesses in the mild-myopia, moderate-myopia, and high-myopia groups were 99.84 ± 4.64 *μ*m, 97.08 ± 5.42 *μ*m, and 95.82 ± 5.75 *μ*m, respectively (*P* < 0.001, Table 2). Average mGCC thickness was thickest in the mild-myopia group, compared with the moderate-myopia group (*P* < 0.001) and the high-myopia group (*P* < 0.001), while there was no statistical difference between the moderate-myopia and high-myopia groups (*P* = 0.114). The mild-myopia group also has the thickest average retinal thickness (Table 2, versus moderate myopia, = 0.037; versus high myopia, *P* = 0.003), but there was no difference of statistics between the moderate-myopia and high-myopia groups (*P* = 0.233). We found that the central subfield thickness were similar among the three groups (*P* = 0.080; Table 2). Comparisons of the parameters among the three groups were shown in Table 2.

To determine potential factors associated with macular vascular density in all subjects, correlation analyses were performed. The variable correlated with both superficial and deep macular vascular densities was MAP (*P* < 0.05, Table 3). In contrast, both superficial and deep macular vascular densities were not correlated with SE or AL in the study subjects (*P* > 0.05, Table 3). The detailed correlation analyses were summarized in Table 3.

**Figure 1:**
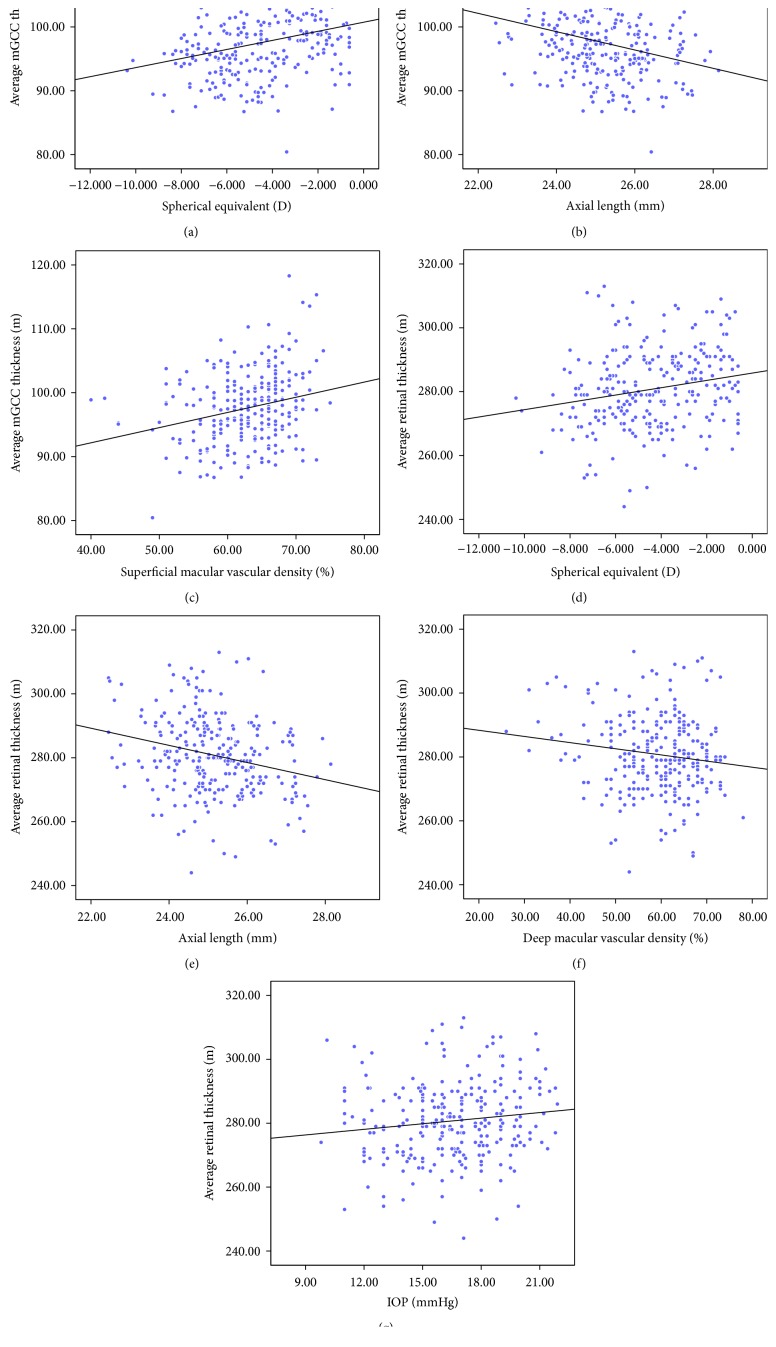
Scatterplots showing correlations between retinal thickness and vascular density in the macular areas. The average mGCC thickness was strongly correlated with spherical equivalent (a), axial length (b), and superficial macular vascular density (c). The average retinal thickness was strongly correlated with spherical equivalent (d) and axial length (e), while it was weakly correlated with deep macular vascular density (f) and IOP (g). mGCC: macular ganglion cell complex; IOP: intraocular pressure.

**Figure 2:**
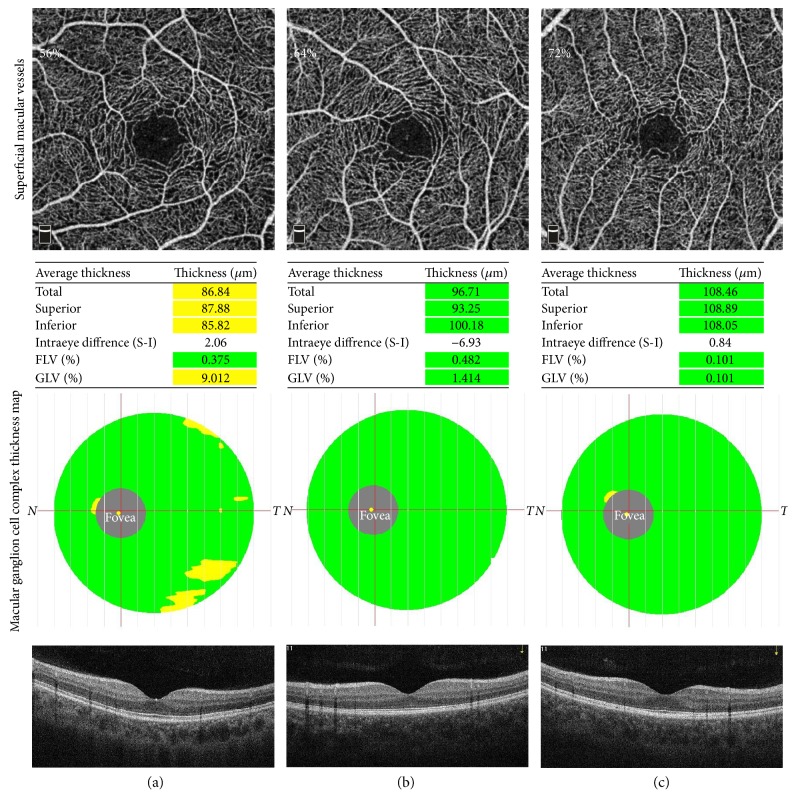
Macular ganglion cell complex thickness map in the eyes with various densities of superficial macular vessels as measured by optical coherence tomography angiography. (a) The superficial macular vascular density is 56%, and the average mGCC thickness is 86.84 *μ*m; (b) the superficial macular vascular density is 64%, and the average mGCC thickness is 96.71 *μ*m; (c) the superficial macular vascular density is 72%, and the average mGCC thickness is 108.46 *μ*m. mGCC: macular ganglion cell complex.

We then performed linear regression analyses to investigate the relationship between vascular density and retinal thickness of the macula. By univariate linear regression analysis, SE, AL, and superficial macular vascular density were revealed to be strongly correlated with average mGCC thickness (all *P* < 0.001; Table 4, Figures 1(a)–1(c)). Multivariate linear regression analysis showed that the association between superficial macular vascular density and average mGCC thickness was independent of AL, and the model revealed that each 100% increase in superficial macular vascular density was associated with a 28.2% increase in average mGCC thickness theoretically (*β* = 0.282, *P* < 0.001; Table 4).  Figure 2 shows that eyes with a greater superficial macular vascular density had thicker mGCCs. Furthermore, the average retinal thickness was strongly correlated with AL and SE, while weakly correlated with IOP at imaging and deep macular vascular density (all *P* < 0.05, Table 4, Figures 1(d)–1(g)). Table 4 shows the detailed results of the linear regression analyses.

Moreover, for those subjects of both eyes included, we performed monocular analyses to further consolidate our results. The left or right eye of one subject was randomly selected with the use of random numbers generated with a seed. The results of monocular analyses were similar to the binocular analyses above. In detail, monocular analyses showed that the superficial macular vascular density was 62.6 ± 6.0% for the mild-myopia group, 63.0 ± 5.6% for the moderate-myopia group, and 64.0 ± 5.4% for the high-myopia group, without significant differences in either measurement (*P* = 0.486); the deep macular vascular density was also similar in the three myopia groups (58.5 ± 9.2% versus 58.6 ± 9.1% versus 61.2 ± 7.8%, *P* = 0.290). Superficial and deep macular vascular densities both had correlations with MAP (*r* = −0.179 and *r* = −0.286, resp.), but not with SE or AL. The standardized regression coefficients of the multivariate linear regression model for mGCC thickness in monocular analysis were as follows: −0.250 (95% CI −0.407–−0.092) for AL; 0.259 (95% CI 0.101–0.417) for superficial macular vascular density.

## 4. Discussion

In this study, we performed a quantitative assessment of macular vascular density using OCTA and compared the macular perfusion among young healthy subjects with varying degrees of myopia. Of note, we found that both superficial and deep macular vascular densities were not significantly different among the three myopia groups and we demonstrated that SE did not influence the macular vascular density in myopic eyes without myopic degeneration or pathologic changes. In addition, we examined whether vascular density was correlated with retinal thickness of the macular regions. The analyses revealed that superficial macular vascular density, as an independent factor, was closely and positively correlated with average mGCC thickness in young myopia.

Before OCTA became widely available, various techniques had been used for retinal blood flow measurement; of these, Doppler imaging was the most applicable for quantitative study. Shimada et al. found that retinal blood flow was significantly decreased in high-myopia compared with emmetropic eyes or mild myopic eyes using laser Doppler velocimetry [30]. Benavente-Pérez and associates demonstrated that high myopes exhibited significantly reduced pulse amplitude and central retinal artery blood velocity [31]. Karczewicz and Modrzejewska also found decreased blood flow in myopia using Doppler ultrasonography [32]. Although vascular density is a sensitive parameter for retinal perfusion but not completely equal to the blood flow detected by Doppler velocimetry, the speed and direction of the blood flow could predominantly affect Doppler velocimetry but could not seriously affect the vascular density detected by OCTA. Furthermore, Doppler imaging is more sensitive to large vessels than the microvasculature, which is a key limitation when measuring retinal vascular networks. Therefore, due to the different equipment used, the conclusions of those previous studies were not highly comparable with our present results.

As technology has advanced, OCTA with SSADA made it possible to provide more detailed morphologic microvascular information in a noninvasive and quantitative way. Retinal vessels, including large vessels and the microvasculature, can be calculated in a defined region in an OCTA image. We used OCTA to quantitatively assess macular vascular density and found no significant differences among mild myopia, moderate myopia and high myopia, which was highly consistent with a prior study by Wang and associates [33]. Furthermore, Mo et al. found no significant decrease in retinal flow density in the macula in high myopic eyes compared with emmetropic eyes [34], which is consistent with our results. Fan et al., in contrast, concluded that more severe myopia was associated with decreased vascular density of the macula [35], but those authors included myopic eyes with peripheral retinal degeneration in their analyses, which indicates that pathologic myopia might influence the retinal perfusion in the macula. The research of Mo et al. confirmed this, finding that macular flow density decreased in pathological myopia compared to those in high myopia and emmetropia [34]. However, Al-Sheikh et al. found that the density of the retinal capillary microvasculature was reduced in eyes with greater myopia among people at an average age of 57 years [36]. Considering that the reduction in macular perfusion is prominent in older subjects, particularly those aged more than 35 to 40 years [37, 38], we focused on subjects in the age range of 18 to 32 years to avoid the effects of the aging process. We demonstrated that SE did not affect the macular vascular density in young myopic eyes without pathologic changes.

In our study, we observed that mGCC thickness was significantly correlated with SE and AL, which is consistent with several previous studies [39–43], and linear regression analyses showed that the superficial macular vascular density, as an independent factor, was strongly associated with the average mGCC thickness. One proposed reason for this observation is that the retinal vascular network supplies abundant nourishment to the retinal tissues [44]. The boundaries of the macular superficial network extended from 3 *μ*m beneath the ILM to 15 *μ*m beneath the IPL, and the mGCC, comprising the retinal nerve fiber layer (RNFL), the ganglion cell layer (GCL), and IPL, is mainly located in the same vertical position [18, 45]. Anatomically, the superficial vasculature, instead of the deep vasculature, mainly nourishes the mGCC. However, Fan et al. found that both superficial and deep macular vascular densities were significantly associated with mGCC thickness [35]. Compared with the study of Fan et al., we included 268 eyes from 138 subjects, about three times as many as the number of their sample size, which might have reduced false-positive rates in our study. Moreover, their inclusion criteria were slightly different from our criteria. Longitudinal studies are needed to clarify the clinical and pathophysiological relevance of the relationship between reduced vessel density and decreased mGCC thickness in myopia.

Furthermore, the results indicated that the average retinal thickness was strongly correlated with AL and SE, which mainly corresponds with results from other studies [46–49] and histological findings of increasing retinal thinning in myopic eyes [50]. Myopization correlates with axial elongation, resulting in biomechanical stretching of the retina, choroid, and sclera, which could cause reduction of the retinal thickness [48]. Besides, average retinal thickness was founded to increase with IOP in young adults, which is consistent with the study of Jin et al. performed in children [51]. The regression analysis showed that average retinal thickness was weakly negatively correlative to deep macular vascular density. It is well known that the human eye is perfused by two extensions of the ophthalmic artery, of which the inner retina is nourished by the retinal vasculature system and the outer retina is supported by the choroidal vascular system [52]; thus, the average retinal thickness is theoretically influenced by both choroidal and retinal vasculature systems. Considering our study's lack of quantitative choroidal vasculature and the small regression coefficient indicating a weak correlation, further studies are needed to confirm the relationship between deep macular vascular density and average retinal thickness.

Although several previous studies have focused on the macular vascular density, this study has certain advantages. To investigate early changes of retinal perfusion and retinal thickness in myopia and establish a macular vascular density database of for young, healthy myopes, we included a fairly large population with normal visual acuity, excluded myopic eyes with signs of myopic degeneration or pathologic changes, used an imaging technique with excellent capillary resolution and reproducibility, and incorporated an automated analysis software. In addition, when investigating the association between the retinal thickness and perfusion of the macula, this study was adjusted for potential confounding factors, such as SE and AL; thus, the conclusions of this study are more reliable.

Nevertheless, this study was limited by its cross-sectional design and the age range of the subjects was relatively small. Since several studies have suggested that age influences macular vascular density [53–55], the narrower age range in this work may confer an advantage by removing age as a potential confounder. Further longitudinal studies with a greater age spectrum might be more informative regarding the retinal structure and blood flow in myopic eyes. Moreover, our quantitative vascular density was not confirmed by another assessment method, although there is no gold standard for vascular density measurements that we could compare to our quantitative data. Impeded by current scanning procedures, the window size was not completely equivalent when measuring macular vascular density, average mGCC thickness, and full retinal thickness. Considering irregular structure of the macula, if new scanning procedures is developed, we could conduct research with the same window size of measurement to further confirm our results.

In conclusion, our study using OCTA found that superficial and deep macular vascular densities were not significantly different among the three myopia groups and demonstrated that SE did not affect macular vascular density in myopic eyes of young adults. In addition, superficial macular vascular density, as an independent factor, was significantly and positively correlated with average mGCC thickness in young myopes. How superficial macular vascular density and mGCC thickness interact with each other remains to be investigated in future studies. OCTA imaging of retinal microvasculature has the potential to become a noninvasive and practical technique for quantitative evaluation and understanding of the underlying mechanisms of various early pathologic changes related to myopia.

## Figures and Tables

**Table 1:** Demographic characteristics among the different myopia groups.

Variables	Mild myopia	Moderate myopia	High myopia	*P*
Number of eyes	81	117	70	
Age (year)	26.1 ± 2.0	26.0 ± 1.6	26.1 ± 1.7	0.744
IOP at imaging (mmHg)	16.51 ± 2.65	16.39 ± 2.81	17.02 ± 2.31	0.271
Spherical equivalent (D)	−1.75 ± 0.72	−4.65 ± 0.84	−7.14 ± 0.94	**<0.001**
Systolic BP (mmHg)	115.31 ± 11.51	114.86 ± 11.68	112.63 ± 10.35	0.297
Diastolic BP (mmHg)	69.02 ± 7.04	68.71 ± 7.75	66.44 ± 6.66	0.059
MAP (mmHg)^†^	84.45 ± 7.21	84.09 ± 8.13	81.84 ± 6.93	0.071
Pulse pressure (mmHg)^‡^	46.28 ± 10.66	46.15 ± 9.38	46.19 ± 8.79	0.996
Heart rate (beats/min)	77 ± 13	79 ± 11	80 ± 12	0.458
Axial length (mm)	24.23 ± 0.94	25.21 ± 0.76	26.15 ± 0.93	**<0.001**
Central corneal thickness (*μ*m)	541.96 ± 37.90	539.37 ± 34.10	532.79 ± 31.33	0.250

Data are expressed as the mean ± SD. SD: standard deviation; IOP: intraocular pressure; BP: blood pressure; MAP: mean arterial pressure. ^†^MAP = 1/3 × systolic BP + 2/3 × diastolic BP; ^‡^pulse pressure = systolic BP − diastolic BP.

**Table 2:** Optical coherence tomography measurements and macular perfusion parameters among the different myopia groups.

Variables	Mild myopia	Moderate myopia	High myopia	*P*
Superficial macular vascular density (%)	62.3 ± 5.7	62.7 ± 5.9	63.8 ± 5.5	0.254
Deep macular vascular density (%)	58.3 ± 9.6	59.2 ± 9.3	60.9 ± 7.9	0.202
Average mGCC thickness (*μ*m)	99.84 ± 4.64	97.08 ± 5.42	95.82 ± 5.75	**<0.001**
Central subfield thickness (*μ*m)	242.21 ± 22.09	248.39 ± 18.09	246.74 ± 17.31	0.080
Average retinal thickness (*μ*m)	283.83 ± 11.49	280.23 ± 11.93	278.09 ± 12.17	**0.011**

Data are expressed as the mean ± SD. mGCC: macular ganglion cell complex.

**Table 3:** Relationships between the clinical variables and macular vascular density.

Variables	Superficial macular vascular density		Deep macular vascular density	
	*r*	*P*	*r*	*P*
Age (year)	−0.058	0.345	−0.023	0.345
IOP at imaging (mmHg)	0.023	0.711	0.032	0.599
Spherical equivalent (D)	−0.088	0.153	−0.095	0.122
MAP (mmHg)	−0.126	**0.039**	−0.217	**<0.001**
Pulse pressure (mmHg)	0.003	0.964	−0.110	0.073
Heart rate (beats/min)	−0.022	0.723	0.010	0.875
Axial length (mm)	0.103	0.093	0.068	0.269
Central corneal thickness (*μ*m)	−0.008	0.890	−0.015	0.813
Average mGCC thickness (*μ*m)	0.249	**<0.001**	−0.041	0.504
Central subfield thickness (*μ*m)	0.095	0.120	−0.074	0.227
Average retinal thickness (*μ*m)	0.031	0.611	−0.145	**0.017**

IOP: intraocular pressure; MAP: mean arterial pressure; mGCC: macular ganglion cell complex.

**Table 4:** Linear regression analysis of factors affecting the average mGCC, central subfield, and average retinal thickness.

Variables included in the model	Average mGCC thickness		Central subfield thickness		Average retinal thickness	
	Beta	*P*	Beta	*P*	Beta	*P*
*Univariate linear regression*						
Age (year)	−0.084	0.170	−0.096	0.119	−0.088	0.150
IOP at imaging (mmHg)	0.066	0.284	−0.074	0.227	0.128	**0.036**
Spherical equivalent (D)	0.284	**<0.001**	−0.108	0.077	0.209	**<0.001**
Axial length (mm)	−0.292	**<0.001**	0.098	0.110	−0.250	**<0.001**
Superficial macular vascular density (%)	0.249	**<0.001**	0.095	0.120	0.031	0.611
Deep macular vascular density (%)	−0.041	0.504	−0.074	0.227	−0.145	**0.017**
*Multivariate linear regression*						
Age (year)						
IOP at imaging (mmHg)					0.128	**0.030**
Axial length (mm)	−0.321	**<0.001**			−0.239	**<0.001**
Superficial macular vascular density (%)	0.282	**<0.001**				
Deep macular vascular density (%)					−0.133	**0.024**

IOP: intraocular pressure; mGCC: macular ganglion cell complex.
